# Coevolution of teaching ability and cooperation in spatial evolutionary games

**DOI:** 10.1038/s41598-018-32292-7

**Published:** 2018-09-20

**Authors:** Shuhua Zhang, Zhipeng Zhang, Yu’e Wu, Yu Li, Yunya Xie

**Affiliations:** Coordinated Innovation Center for Computable Modeling in Management Science, Tianjin University of Finance and Economics, Tianjin, 300222 China

## Abstract

Individuals with higher reputation are able to spread their social strategies easily. At the same time, one’s reputation is changing according to his previous behaviors, which leads to completely different teaching abilities for players. To explore the effect of the teaching ability influenced by reputation, we consider a coevolutionary model in which the reputation score affects the updating rule in spatial evolutionary games. More precisely, the updating probability becomes bigger if his/her partner has a positive reputation. Otherwise, the updating probability becomes smaller. This simple design describes the influence of teaching ability on strategy adoption effectively. Numerical results focus on the proportion of cooperation under different levels of the amplitude of change of reputation and the range of reputation. For this dynamics, the fraction of cooperators presents a growth trend within a wide range of parameters. In addition, to validate the generality of this mechanism, we also employ the snowdrift game. Moreover, the evolution of cooperation on Erdős-Rényi random graph is studied for the prisoner’s dilemma game. Our results may be conducive to understanding the emergence and sustainability of cooperation during the strategy adoptions in reality.

## Introduction

The prevalence of cooperative or altruistic behaviors is a ubiquitous phenomenon among selfish and unrelated agents, ranging from biological spheres to social communities^1–4^. Therefore, understanding the evolution of cooperation is one of the enduring conundrums of behavior sciences^5–7^. This has attracted plenty of attentions from biologists, economists and physicists. Evolutionary game theory is one of the fruitful frameworks, based on the so-called social dilemmas in which social conflicts are analogous to the competition among individuals^8–10^, to investigate this problem. Among these social dilemmas, the prisoner’s dilemma game (PDG) and the snowdrift game (SDG) are the most prominent metaphors for pairwise interactions^11^.

In the original game, two players (agents) make a decision simultaneously between cooperation (*C*) and defection (*D*). If both players cooperate, they will get the reward *R* equally, but only the punishment *P* when two defectors encounter. On the contrary, the defector could get the highest temptation *T* and the cooperator only obtains the sucker’s payoff *S* if two players have different actions. For the PDG, the payoff must satisfy the necessary rankings *T* > *R* > *P* > *S* and 2*R* > (*T* + *S*). Mutual defection is the solely stable state or Nash equilibrium of the game, therefore, it is the rational choice. In other words, although mutual cooperation yields the most collective payoff, rational players always defect regardless of what the opponent chooses^12^. For the SDG, the payoff ranking is *T* > *R* > *S* > *P*. This minor variation results in a significant change with the optimal strategy. Namely, there are two Nash equilibria in this game: defect when your opponent cooperates and cooperate when your opponent defects^13^. According to the Nash equilibria of the games, it is easy to see that SDG is more supportive for cooperation than the PDG. However, the Nash equilibria of the above two classical games are contradictory to the fact that cooperation is observed in nature widely. Consequently, some unknown mechanisms are maintaining cooperation in reality definitely.

Over the past decades, various specific mechanisms have been proposed to understand the emergence and maintenance of cooperation in many disciplines. Paradigmatic examples include kin selection^14^, direct and indirect reciprocity^15–17^, group selection^18^, noise^19,20^, extortion strategies^21^, reward and punishment^22–25^. Furthermore, some effective strategies are also used, such as the tit-for-tat^26,27^ and win-stay lose-shift^28–30^. In particular, spatial structure^31^ has been identified as one of the most effective factors to enhance cooperation. In spatial evolutionary game, players only interact with their nearest neighbors on a regular lattice. Cooperators can resist exploitation of defectors through forming clusters, which can protect those cooperators that are located in the interior of clusters. Following this pioneering work, a great number of promoting mechanisms have been studied. See, for example, the survey articles^32,33^. Complex networks, having a similar connectivity distribution with complex systems in reality, e.g., air transportation networks and the Internet, provide a uniform framework to understand the common cooperation behaviors^34–37^.

Teaching activity^38,39^ is an important process in the evolution of cooperation, which refers to the influence or reproduction rate of individuals. Players with high influence are more likely to reproduce than individuals with low influence, i.e., they have a higher teaching ability. In previous works^38,39^, teaching ability is a control variable, which is unchanged during the evolution. However, teaching ability is changing continuously in reality, as a consequence, we adopt a coevolutionary model in this paper. In addition, it is not hard to imagine that players who have higher reputation are able to spread their social strategies easily. For example, the companies will get higher and higher reputation if they always complete the production tasks on time on the basis of the contracts with other enterprises. Then, more and more firms are not only inclined to cooperate or deal with them, but also more likely to refer to and imitate their way of operational management or technologies. On the contrary, other companies are not willing to imitate them. Therefore, the logical assumption using reputation score to symbolize the teaching ability is reasonable. Reputation represents a class of individual information which is about one’s past behaviors. It will change according to one’s past behaviors. It has promoted the evolution of cooperation effectively in games of indirect reciprocity. As a classically theoretical model of reputation, image scoring has been studied extensively in which cooperative behaviors increase reputations and defective actions decrease the score by one unit^40^. It has been proved that cooperation can be enhanced evidently with the aid of reputation. Migration based on the reputation has been introduced into the spatial PDG^41^. Individuals can adjust their partnerships on the basis of local information about reputation^42^. The time scale of selection and updating will change if reputation is introduced^43,44^. Some coevolutionary models about time scale and cooperation are employed in previous works^45,46^ and the results show that cooperation can be promoted when an individual with a high payoff holds a successful strategy for a longer time. In the present paper, strategy updating and teaching ability have the same time scale. In addition, cognitive ability based on reputation is also studied, inferring that reputation mechanism can be seen as a universally applicable promoter of cooperation, which works on various interaction networks and in different types of evolutionary game^47–51^. However, one can not ignore partners’ reputation (teaching ability) when he updates his strategy because reputation includes a lot of information about the partner. Obviously, one’s teaching ability could affect partner’s decision directly. Generally speaking, one is perhaps more likely to adopt partner’s strategy with a good reputation and excludes the one with a bad reputation. For example, virtuous people usually spread their minds easily in reality. This form of connection between reputation and partner’s teaching ability is not studied in previous work. Therefore, a more realistic scenario will acknowledge that a player will make a decision by taking the teaching ability into consideration.

Based on the above facts, in the present paper, we propose a modified updating rule incorporated with partner’s reputation to describe the teaching ability. It is assumed that individuals acquire reputation without extra cost because reputation information can spread among neighbors by gossip. The PDG and SDG are employed to model social dilemmas, in which interactions are driven by complex topologies. In this paper, we consider the regular lattice (the neighborhood setups are the von Neumann neighborhood or the Moore neighborhood, in other words, the degree *k* is equal to 4 or 8 for each vertex, respectively) and the Erdős-Rényi (ER) random graph. For the ER random graph, the average degree $\bar{k}$ is equal to 4. Simulation results show that a higher level of cooperation appears when teaching ability is in effect during the decision making process.

## Results

Teaching ability, represented by reputation score *R*_*i*_, is introduced into the strategy updating rule to explore its influences on the emergence of cooperative behavior in spatial evolutionary games. The influences of one’s teaching ability change during the evolution of games. The change amplitude of *R*_*i*_ is *δ* (>0) every time. That is to say, choosing cooperation for player *i* will lead to *R*_*i*_ increases by *δ*. Otherwise, it decreases by *δ*. Additionally, *R*_*i*_ ∈ [−*α*, *α*] (*α* > 0), which means that the value of reputation has a saturation effect whether it is good or bad. Reputation score *R*_*i*_ has an important effect on the strategy updating for player *i*. From a qualitative point of view, the probability of strategy updating becomes more bigger if *R*_*i*_ is positive. Instead, it turns smaller. Furthermore, *δ*/*α* is the fluctuation ratio of reputation. It represents the intensity of teaching ability. In the following results, we set the size of the regular lattice from 100 × 100 to 200 × 200. The size of the ER random graph is 10000. And Monte Carlo (MC) simulation is repeated for 61000 times. The details of interactions between agents and their corresponding payoffs are summarized in the *Methods* section.

**Figure 1:**
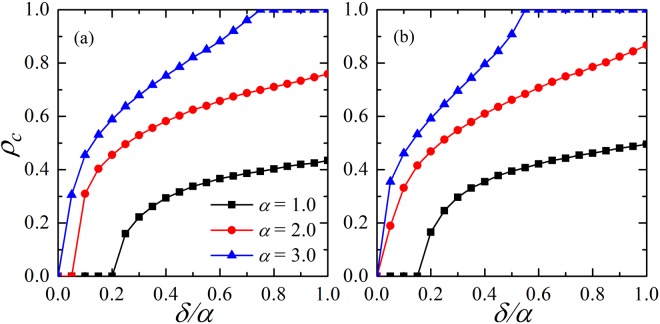
Concentration of cooperators *ρ*_*c*_ *vs* the quotient of *δ*/*α* in the prisoner’s dilemma game, *α* = 1.0, 2.0 and 3.0, respectively. For both panels, the results are obtained on a 200 × 200 regular lattice. The results in panel (a) are acquired in the von Neumann neighborhood, while the results are according to the Moore neighborhood in panel (b). As the value of *δ*/*α* increases, the effect of teaching ability becomes obvious. Parameter *b* = 1.15.

We start by examining the effect of the new strategy adoption rule on the persistence of cooperation. As shown in Fig. 1, two different sizes of neighbors are compared to analyze the impact of strategy selection on the evolution of cooperation on the regular lattice (200 × 200) with periodic boundary conditions. More concretely, panels (a) and (b) are corresponding to the results for the von Neumann neighborhood and the Moore neighborhood^52^, respectively.

*δ* = 0 means that the model degenerates to the traditional version and the normalized payoff difference (*P*_*i*_ − *P*_*j*_)/*k* is the sole determinant factor for strategy updating. According to the previous research^31^, cooperators could form clusters to prevent defectors from invasion, which is called the network reciprocity. However, the cooperators located on the edge of the cooperative clusters are prone to revolting as the value of *b* increases, which results in the dissolution of cooperative clusters eventually (i.e., in the two traditional cases, the values of *b*, which make the cooperators vanish, are less than 1.1).

As shown in panels (a) and (b), once *δ* > 0, the evolution of the whole system becomes totally different because the teaching ability is considered. The normalized payoff difference (*P*_*i*_ − *P*_*j*_)/*k* and the teaching ability *R*_*i*_ decide the strategy updating at the same time. Moreover, *b* is fixed to 1.15. For each curve in Fig. 1, the fraction of cooperation *ρ*_*c*_ monotonically increases with the increasing of the fluctuation ratio of reputation *δ*/*α*. Although the temptation to defect *b* is high, this mechanism can guide the players to select cooperation effectively so that cooperators survive in the system. It can be observed that cooperators could dominate the whole network in some cases. The introduction of teaching ability makes individuals update strategy depends on the normalized payoff difference and the teaching ability. And this updating rule seems to be reasonable and makes cooperation become the dominant strategy. Furthermore, the range of reputation *α* has a great impact on the evolution of cooperation. Obviously, the speed and intensity of cooperators appearing and spreading in the *α* = 3 condition are more remarkable than those of *α* = 1 or *α* = 2. However, the gap between the two curves of *α* = 2 and *α* = 3 is smaller than that between *α* = 1 and *α* = 2. This suggests that the effect of the range of reputation will not increase immensely. The above results clearly indicate that the evolution of cooperation is greatly promoted under the newly introduced mechanism.

**Figure 2:**
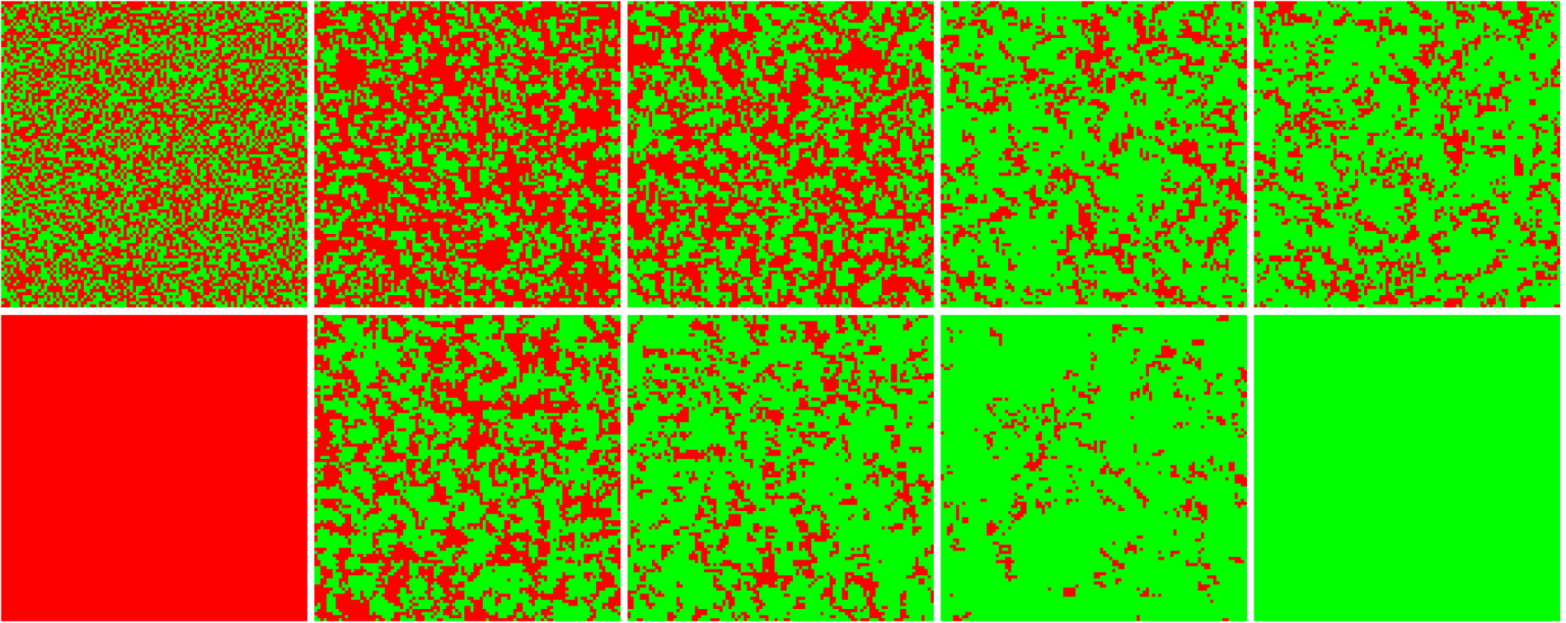
Typical strategy distribution of cooperators (green) and defectors (red) on a 100 × 100 square lattice. Snapshots are given at *t* = 0, 5, 10, 100, 60000 steps in the upper row. The lower panels are the distribution of strategies at the 60000*th* step. The amplitudes of change of reputation are *δ* = 0, *δ* = 0.75, *δ* = 1.5, *δ* = 2.25 and *δ* = 3.0 from left to right, respectively. The parameters are *b* = 1.17, *α* = 3 and *k* = 4.

It remains interesting to elucidate how this new mechanism promotes cooperation. To provide answers, we show some characteristic snapshots on a 100 × 100 square lattice (the von Neumann neighborhood) in Fig. 2 (the green and the red represent the cooperators and the defectors, respectively). The parameter *b* is given by a constant term in all snapshots (*b* = 1.17). First, looking at the upper row, the snapshots are given for *t* = 0, 5, 10, 100, 60000. As shown, cooperators and defectors uniformly scatter all over the lattice initially. As described earlier, cooperators will die out in the traditional version in this condition. However, the evolution is obviously different once the teaching ability (*δ* = 1.5 and *α* = 3.0) is incorporated. Compared with the traditional case, all players take more information (the teaching ability) into account when they make a decision. Cooperative clusters could protect cooperators granted that the value of *b* is high. Many a cooperator is able to survive at the stable stage even though the fraction of cooperators will fall at the beginning of the evolution.

To compare with Fig. 1, we also explore the distribution of strategies at the 60000*th* Monte Carlo step (MCS) in the lower panels of Fig. 2. The parameters are *δ* = 0, *δ* = 0.75, *δ* = 1.5, *δ* = 2.25, *δ* = 3.0 from left to right and *α* = 3.0. The other configurations are consistent with the upper row. For *δ* = 0, cooperators still can not survive because the selection intensity is not enough to resist the temptation to defection. With the increasing of *δ*, many big and compact clusters form steadily when the mechanism works. For example, the territory of the defectors becomes more and more smaller when *δ* increases from 0 to 3. The increased teaching ability means that some information except the payoff becomes more and more important for individuals. Such a consideration is reasonable, e.g., one will consider a lot of things besides the profit when he makes a decision. These results illustrate that this mechanism can facilitate the network reciprocity remarkably. Based on this fact, it is not hard to understand that cooperators can dominate the whole system when *δ* reaches the maximum at the same condition. The simulated phenomena imply that the cooperators can survive or even thrive owing to the consideration of appropriate teaching ability.

**Figure 3:**
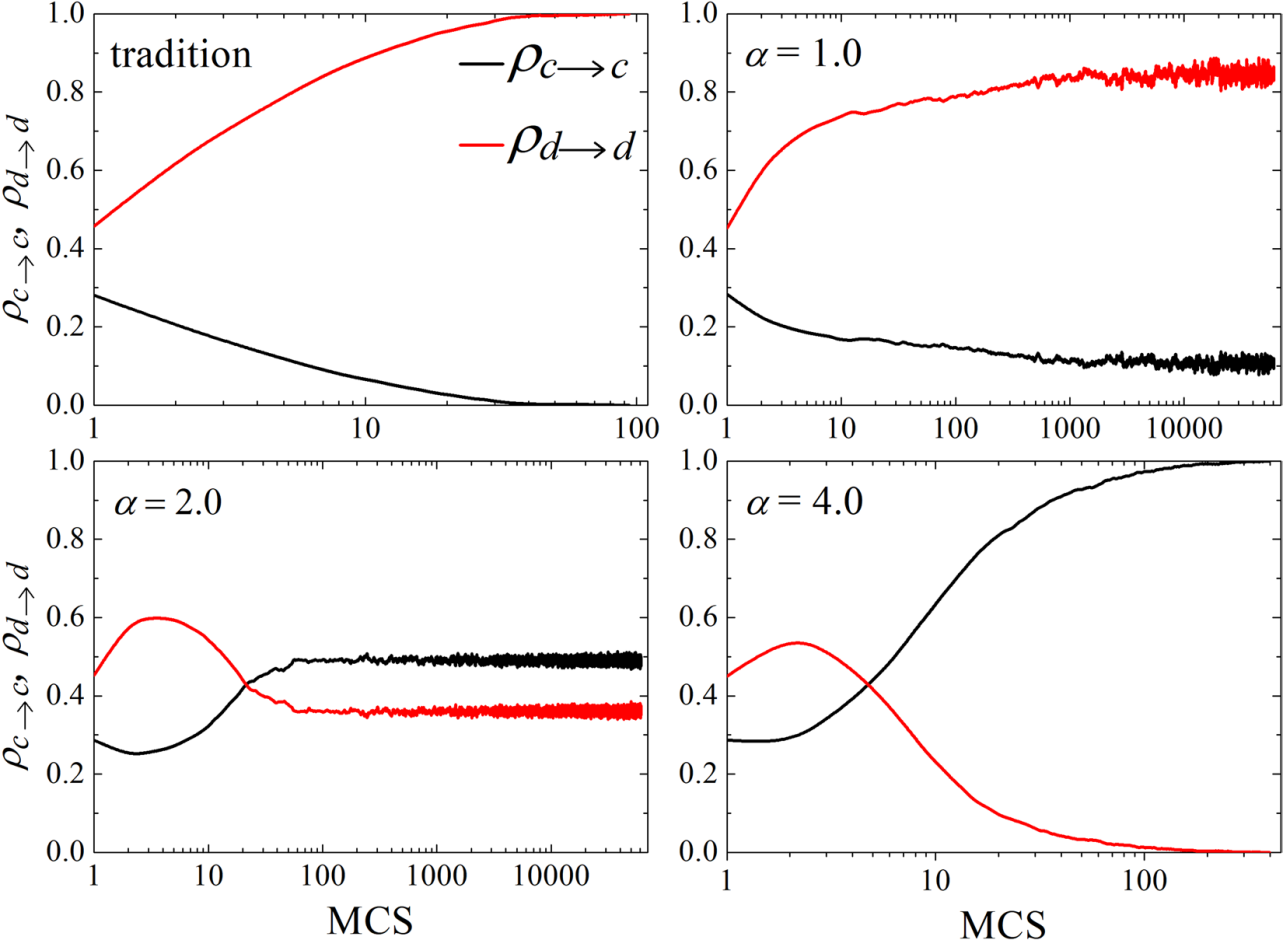
Time evolution of the strategy retention rate *ρ*_*c*(*d*)→*c*(*d*)_. *ρ*_*c*→*c*_ represents the rate that cooperators continue to cooperate and *ρ*_*d*→*d*_ denotes the rate that defectors continue to retain defective strategy. *δ*/*α* is fixed to 0.75 and the panels are corresponding to *α* = 1.0,2.0,4.0 except the traditional case. With the increasing of *α*, the readiness to cooperate increases gradually. The depicted results are obtained on a 200 × 200 square lattice, with the parameter *k* = 4 and *b* = 1.2.

Note that there are two major factors to affect the probability of strategy updating from the above analyses: the normalized payoff difference and the value of reputation. Therefore, it is necessary to study the individual’s preference for strategies. What follows is an observation about the temporal traits of strategy retention rate. As shown in Fig. 3, the parameter *b* is fixed to 1.2 in every panel and *δ*/*α* = 0.75 except the traditional case. Therein, *ρ*_*c*→*c*_ represents the rate that cooperator is still a cooperator between two rounds. Analogously, *ρ*_*d*→*d*_ is the rate that defectors retain defective strategies along with time. For the traditional case, *ρ*_*c*→*c*_ reaches to 0 and *ρ*_*d*→*d*_ becomes 1 fast since the temptation to defect is high. Consequently, cooperators become extinct soon in the background of the payoff of defectors being more than that of cooperators. However, cooperators could occupy a certain territory when teaching ability (*α* = 1.0) is taken into consideration. For *α* = 2.0, *ρ*_*c*→*c*_ first drops and the trend of *ρ*_*d*→*d*_ is opposite completely, which proves that the overall atmosphere is still unfavorable for the persistence of cooperation even though with the help of different teaching abilities in the early stage. After that stage, *ρ*_*c*→*c*_ fast upward pulls and *ρ*_*d*→*d*_ falls rapidly and *ρ*_*c*→*c*_ even exceeds *ρ*_*d*→*d*_. Since the cooperation strategy is more likely becoming reference selection, so it is not hard to understand that the evolution of cooperation widely spreads in Fig. 2. For *α* = 4.0, the evolution trend is similar to *α* = 2.0. However, the gap between two curves becomes bigger and bigger and the number of retaining cooperation will increase to 1. This process means that the prevalence of cooperation is positively related to the value of *α*. We could draw a conclusion that the incorporation of teaching ability influenced by reputation adjusts the microscopic preference of players and accelerates the dissemination of cooperation based on these results. These promoting effects are consistent with the aforementioned results.

**Figure 4:**
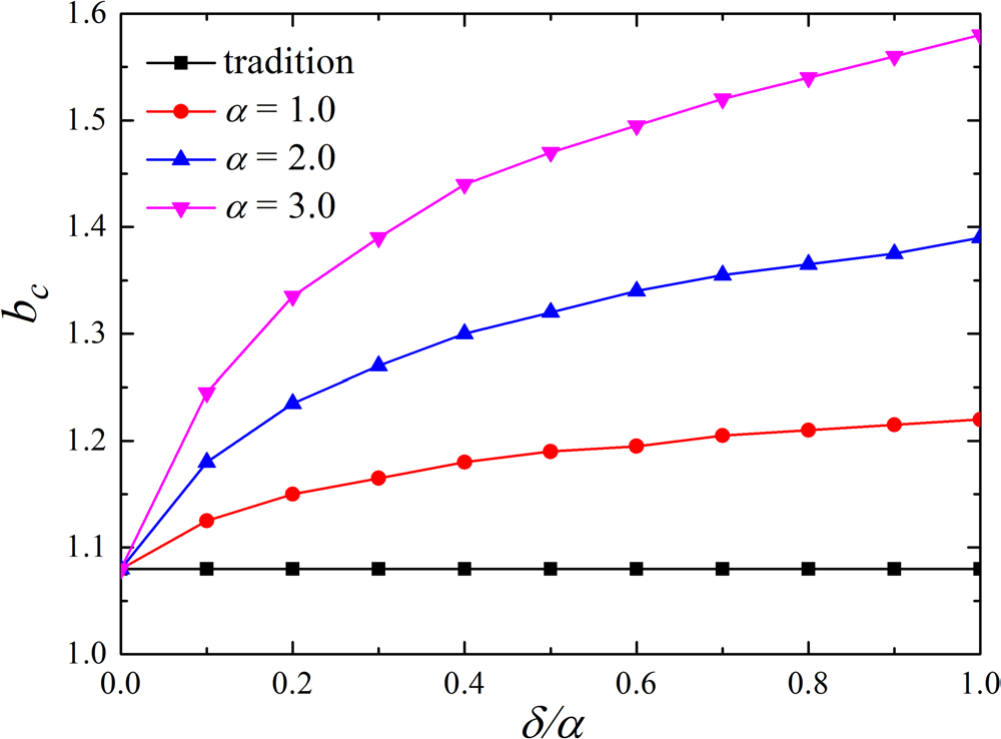
Critical threshold values *b* = *b*_*c*_ to make the evolution to the pure *D* phase (extinction of cooperators). It can be observed that *b*_*c*_ increases monotonically with the increasing of the fluctuation ratio of reputation *δ*/*α*. These results shows that the space occupied by cooperators is enlarged. The simulation is executed on a 200 × 200 square lattice and the neighborhood setup is the von Neumann neighborhood.

Besides, it deserves to consider how the critical threshold value of *b*_*c*_ changes with the fluctuation ratio parameter *δ*/*α*. Fig. 4 is the simulation results on a 200 × 200 square lattice (*k* = 4). As shown in Fig. 4, *b*_*c*_ denotes the threshold for cooperators to die out. For the traditional version, we could see a straight line (black) at the bottom of Fig. 4. It indicates that all players only care about payoffs so that defection becomes the rational choice. However, it can be observed that *b*_*c*_ increases monotonically from left to right for other three curves, which means that the space of cooperators living is enlarged as *δ*/*α* increases. Those players with good reputation restrain selfish agents from adopting defection. For example, the cooperators are located in the edge of clusters in Fig. 2 are more loyal to cooperation. This result fully explains that the new idea could promote the survival of cooperators among selfish players.

**Figure 5:**
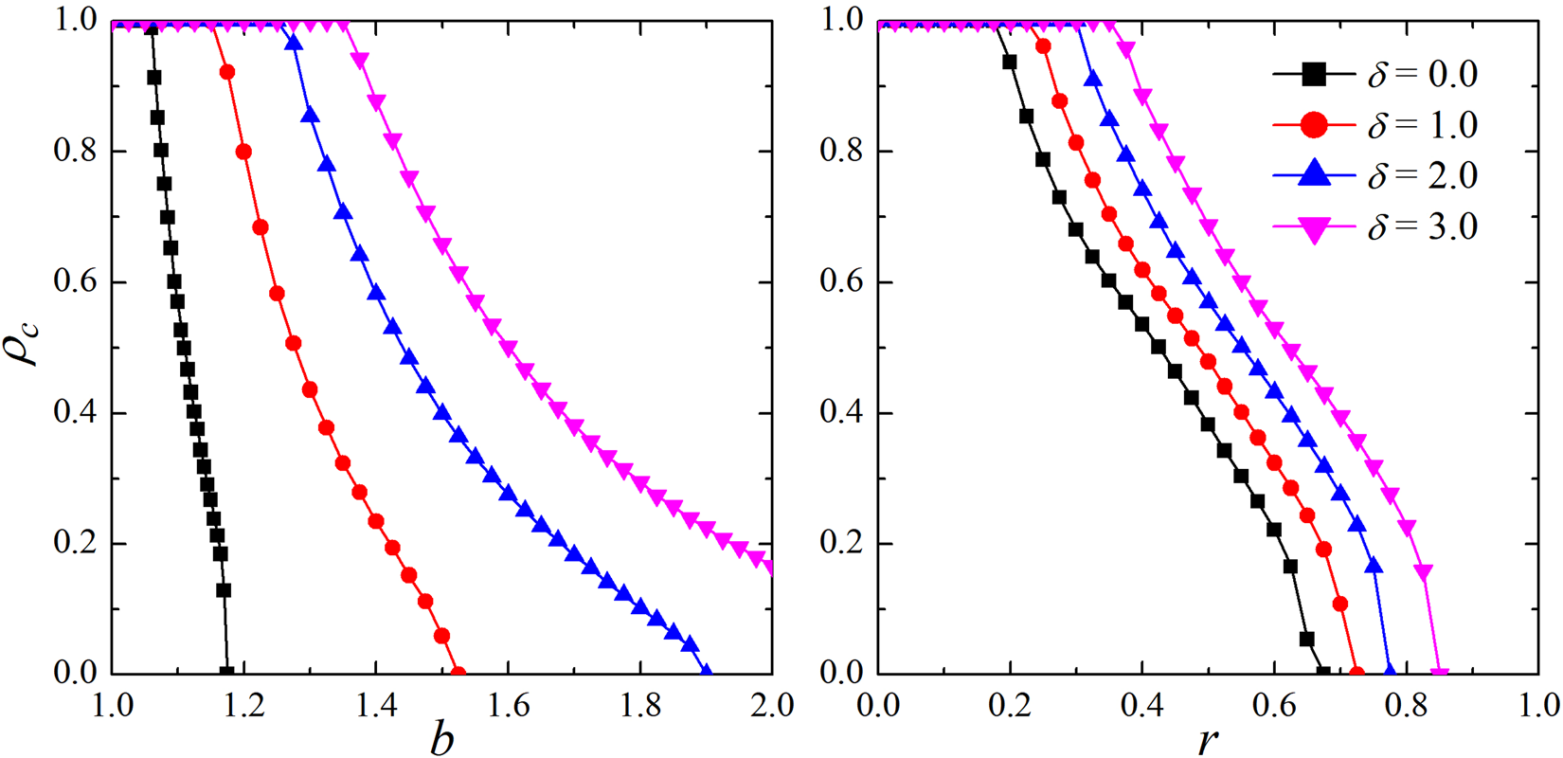
Left panel: fraction of cooperators *ρ*_*c*_ in dependence on the parameter *b* for different values of *δ* in the prisoner’s dilemma game on the ER graph. It has *N* = 10^4^ nodes and its average degree is $\bar{k}$ = 4. Right panel: fraction of cooperators *ρ*_*c*_ of the snowdrift game in dependence of the parameter *r* for different values of *δ* on the regular lattice (size of 200 × 200 and the degree *k* = 4).

Lastly, it is worth exploring the robustness and generality of the above observations by means of different networks and evolutionary game models. Here we set *δ*/*α* = 1 for all curves in Fig. 5. Our MC simulation results in the left panel are about prisoner’s dilemma game on the ER random graph. The network has the same average degree (i.e. $\bar{k}$ = 4) and size (*N* = 10^4^ nodes) with regular lattice. As shown, a virtually promotion effect on the evolution of cooperation can be observed compared with the traditional version (*δ* = 0). The evolution of cooperation is strengthened effectively with the increasing of *δ* in the PDG, which is qualitatively consistent with the results obtained on the regular network. As an example, the critical value *b*_*c*_ has exceeded 2.0 when *δ* = 3.0, which implies that the cooperators can survive or even thrive within a large range of *b*’s values. For the right panel of Fig. 5, it depicts the fraction of cooperators *ρ*_*c*_ of SDG on the regular network (200 × 200 and *k* = 4) depend on the parameter *r*. Likewise, cooperation is enhanced obviously. Anyway, these results support the fact that teaching ability influenced by reputation is a universally effective way to sustain and promote cooperation, regardless of the form of the underlying game and interaction network.

## Discussion

In sum, we have proposed a coevolutionary model to investigate the impact of teaching ability influenced by reputation on the evolution of cooperation in spatial evolutionary games. This model emphasizes the relevance of strategy adoption and teaching ability when human behaviors are modeled. This form of strategy adoption illustrates that the players with high reputation can spread their strategies easily and vice versa. There is no doubt that this coevolution process conforms to the real situation compared to the traditional case. Numerical simulation implies that the amplitude of change of reputation *δ* and reputation’s range *α* have a significant impact on the persistence of cooperation. More cooperative clusters appear easily under this updating dynamics. Those players with good reputation restrain selfish agents from adopting defection strategy. In addition, the robustness of the enhancement effect is checked on ER graph for the prisoner’s dilemma game. The promoting effects are confirmed in the snowdrift game as well. The aforementioned results may illustrate that this mew mechanism has a certain degree of university because it works effectively for the prisoner’s dilemma game and the snowdrift game on two kinds of networks (the regular square lattice and the ER random graph). This work may be conductive to understanding the cooperative behaviors in complex economics as well as human society.

## Methods

In this work, evolutionary PDG and SDG are employed to explore the role of teaching ability influenced by reputation, in which every player occupies a vertex of the underlying networks. For testing the robustness of the impact of this newly introduced mechanism on the evolution of cooperation, different networks topologies including regular lattice and Erdős-Rényi (ER) random graph are taken into consideration. For simplicity but without loss of generality, here we consider a so-called weak PDG^31^, which is characterized with the temptation to defect *T* = *b*, mutual cooperation *R* = 1, the punishment for the mutual defection *P* = 0 and the suckers’ payoff *S* = 0. Therefore, it is not hard to see that the outcome of the game is only dependent on the parameter *b*. In addition, 1 < *b* < 2 quantifies the temptation to defect and represents the advantage of defectors over cooperators. For the SDG, the rescaled payoffs are *T* = 1 + *r*, *R* = 1, *S* = 1 − *r* and *P* = 0, where 0 < *r* < 1 represents the so-called cost-to-benefit ratio and payoffs still satisfy the ranking *T* > *R* > *S* > *P*. Initially, each individual *i* is designed as a cooperator (*s*_*i*_ = *C*) or a defector (*s*_*i*_ = *D*) with equal probability and is given a reputation score coefficient *R*_*i*_ as well. To avoid the preferential influence, we set *R*_*i*_ = 0 before the game. Reputation is important in human society. It reflects one’s history information or status and is accessed by all members in his/her community. Individuals with different reputation scores have different influences on the players interacting with them. As a consequence, we use the reputation score to symbolize the teaching ability. Moreover, it is assumed that reputation spreads among neighbors by gossip and is evaluated under the simple protocol below without cost.

The game is simulated with the following Monte carlo (MC) simulation procedures: firstly, player *i* gets his total payoff *P*_*i*_ by playing the game with his nearest neighbors. Next, player *i* will choose a neighbor *j* randomly from its neighbors as the reference target, who acquires payoff *P*_*j*_ in the same way. Last, all agents synchronously update their strategies according to the following probability:

1 $$Pro{b}_{i}=\frac{1}{1+\exp [({P}_{i}-{P}_{j})/kK-{R}_{j}]},$$

where *K* represents the intensity of selection. Without loss of generality, we set *K* to be 0.1 in this paper if not directly stated^53^. Player *i* will adopt *j*’s strategy relying on the normalized payoff difference (e.g. (*P*_*i*_ − *P*_*j*_)/*k*) and *R*_*j*_. The degree of player *i* is *k*. It is noted that each player has a chance to adopt one of their neighbors’ strategies once on average during one full MC simulation. As mentioned above, here we assume that players have local information about his nearest neighbors. As a consequence, each neighbor’s reputation is known to the focal player and *R*_*j*_ could affect *Prob*_*i*_ directly. Furthermore, it is more practical that reputation is changing with the evolution. Here we assume that *δ* > 0 symbolizes the amplitude of change of reputation. That is to say, *R*_*i*_ increases by *δ* when *i* is a cooperator and *R*_*i*_ decreases by *δ* when *i* chooses defection. Additionally, *R*_*i*_ ∈ [−*α*, *α*](*α* > 0), which means that the value of reputation has a saturation effect whether it is good or bad and *δ*/*α* is the fluctuation ratio of reputation. According to formula (1), player *i* prefers to adopt player *j*’s strategy if *R*_*j*_ > 0. Instead, player *i* has an attitude of exclusion for player *j* if he is notorious. This simple design describes the influence of teaching ability on strategy adoption effectively.

Ultimately, a whole Monte Carlo step (MCS) is finished if the above-mentioned fundamental procedures are implemented. For the regular lattice, the results of MC simulation presented in the *Results* are obtained on populations comprising 100 × 100 up to 200 × 200 agents. And the neighborhood setups are the von Neumann neighborhood or the Moore neighborhood, namely, the degree *k* is equal to 4 or 8 for each agent, respectively. For the ER random graph, the size *N* and the average degree $\bar{k}$ are *N* = 10^4^ and $\bar{k}$ = 4, respectively. Additionally, the fraction of cooperators *ρ*_*c*_ is acquired by averaging the last 1000 full MCS of the total 61000 and the final results are averaged over 10–20 independent runs to guarantee the accuracy.

## References

[1] Jain S, Krishna S (2001). A model for the emergence of cooperation, interdependence and structure in evolving networks. Proc. Natl. Acad. Sci. USA.

[2] Ohtsuki H, Hauert C, Lieberman E, Nowak MA (2006). A simple rule for the evolution of cooperation on graphs and social networks. Nature.

[3] Brandt H, Hauert C, Sigmund K (2003). Punishment and reputation in spatial public goods games. Proc. R. Soc. Lond. B.

[4] Xia CY, Meng XK, Wang Z (2015). Heterogeneous coupling between interdependent lattices promotes the cooperation in the prisoner’s dilemma game. PloS ONE.

[5] Nowak MA (2006). Five rules for the evolution of cooperation. Science.

[6] Santos FC, Santos MD, Pacheco JM (2008). Social diversity promotes the emergence of cooperation in public goods games. Nature.

[7] Wu Y, Zhang Z, Chang S (2017). Effect of self-interaction on the evolution of cooperation in complex topologies. Physica A.

[8] Wu Y, Chang S, Zhang Z, Deng Z (2017). Impact of social reward on the evolution of the cooperation behavior in the complex networks. Sci. Rep..

[9] Perc M (2006). Coherence resonance in a spatial prisoner’s dilemma game. New J. Phys..

[10] Wang Z, Wang L, Yin ZY, Xia CY (2012). Inferring reputation promotes the evolution of cooperation in spatial social dilemma games. PLoS ONE.

[11] Doebeli M, Hauert C (2005). Models of cooperation based on the prisoner’s dilemma and the snowdrift game. Ecol. Lett..

[12] Gómez-Gardeñes J, Reinares I, Arenas A, Floría LM (2012). Evolution of cooperation in multiplex networks. Sci. Rep..

[13] Hauert C, Doebeli M (2004). Spatial structure often inhibits the evolution of cooperation in the snowdrift game. Nature.

[14] Hamilton WD (1964). The genetical evolution of social behaviour. ii. J. Theor. Biol..

[15] Trivers RL (1971). The evolution of reciprocal altruism. Q. Rev. Biol..

[16] Panchanathan K, Boyd R (2004). Indirect reciprocity can stabilize cooperation without the second-order free rider problem. Nature.

[17] Chen X, Schick A, Doebeli M, Blachford A, Wang L (2012). Reputation-based conditional interaction supports cooperation in well-mixed prisoner’s dilemmas. PloS ONE.

[18] Traulsen A, Nowak MA (2006). Evolution of cooperation by multilevel selection. Proc. Natl. Acad. Sci. USA.

[19] Vukov J, Szabó G, Szolnoki A (2006). Cooperation in the noisy case: prisoner’s dilemma game on two types of regular random graphs. Phys. Rev. E.

[20] Szolnoki A, Vukov J, Szabó G (2009). Selection of noise level in strategy adoption for spatial social dilemmas. Phys. Rev. E.

[21] Mao Y, Xu X, Rong Z, Wu ZX (2018). The emergence of cooperation-extortion alliance on scale-free networks with normalized payoff. EPL.

[22] Li X (2018). Punishment diminishes the benefits of network reciprocity in social dilemma experiments. Proc. Natl. Acad. Sci. USA.

[23] Jiménez R, Lugo H, Cuesta JA, Sánchez A (2008). Emergence and resilience of cooperation in the spatial prisoner’s dilemma via a reward mechanism. J. Theor. Biol..

[24] Helbing D, Szolnoki A, Perc M, Szabó G (2010). Punish, but not too hard: how costly punishment spreads in the spatial public goods game. New J. Phys..

[25] Wang Z, Xia CY, Meloni S, Zhou CS, Moreno Y (2013). Impact of social punishment on cooperative behavior in complex networks. Sci. Rep..

[26] Imhof LA, Fudenberg D, Nowak MA (2007). Tit-for-tat or win-stay, lose-shift?. J. Theor. Biol..

[27] Baek SK, Kim BJ (2008). Intelligent tit-for-tat in the iterated prisoner’s dilemma game. Phys. Rev. E.

[28] Nowak MA, Sigmund K (1993). A strategy of win-stay, lose-shift that outperforms tit-for-tat in the prisoner’s dilemma game. Nature.

[29] Chen X, Fu F, Wang L (2008). Promoting cooperation by local contribution under stochastic win-stay-lose-shift mechanism. Physica A.

[30] Amaral MA, Wardil L, Perc M, Da Silva JK (2016). Stochastic win-stay-lose-shift strategy with dynamic aspirations in evolutionary social dilemmas. Phys. Rev. E.

[31] Nowak MA, May RM (1992). Evolutionary games and spatial chaos. Nature.

[32] Perc M, Gómez-Gardeñes J, Szolnoki A, Floría LM, Moreno Y (2013). Evolutionary dynamics of group interactions on structured populations: a review. J. R. Soc. Interface.

[33] Perc M, Szolnoki A (2010). Coevolutionary games–a mini review. BioSystems.

[34] Poncela J, Gómez-Gardeñes J, Floría LM, Moreno Y (2007). Robustness of cooperation in the evolutionary prisoner’s dilemma on complex networks. New J. Phys..

[35] Du WB, Cao XB, Liu RR, Wang Z (2012). Effects of inertia on evolutionary prisoner’s dilemma game. Comm. Theo. Phys..

[36] Wu Y, Zhang B, Zhang S (2017). Probabilistic reward or punishment promotes cooperation in evolutionary games. Chaos Solitons & Fractals.

[37] Cardillo A, Gómez-Gardeñes J, Vilone D, Sánchez A (2010). Coevolution of strategies and update rules in complex prisoner’s dilemma networks. New J. Phys..

[38] Szolnoki A, Szabó G (2007). Cooperation enhanced by inhomogeneous activity of teaching for evolutionary prisoner’s dilemma games. EPL.

[39] Szolnoki A, Perc M, Szabó G (2008). Diversity of reproduction rate supports cooperation in the prisoner’s dilemma game on complex networks. Eur. Phys. J. B.

[40] Nowak MA, Sigmund K (1998). Evolution of indirect reciprocity by image scoring. Nature.

[41] Cong R, Wu B, Qiu Y, Wang L (2012). Evolution of cooperation driven by reputation-based migration. PloS ONE.

[42] Fu F, Hauert C, Nowak MA, Wang L (2008). Reputation-based partner choice promotes cooperation in social networks. Phys. Rev. E.

[43] Wu Z-X, Rong Z, Holme P (2009). Diversity of reproduction time scale promotes cooperation in spatial prisoner’s dilemma games. Phys. Rev. E.

[44] Rong Z, Wu Z-X, Hao D, Chen MZQ, Zhou T (2015). Diversity of timescale promotes the maintenance of extortioners in a spatial prisoner’s dilemma game. New J. Phys..

[45] Rong Z, Wu Z-X, Wang W-X (2010). Emergence of cooperation through coevolving time scale in spatial prisoner’s dilemma. Phys. Rev. E.

[46] Rong Z, Wu Z-X, Chen G (2013). Coevolution of strategy-selection time scale and cooperation in spatial prisoner’s dilemma game. EPL.

[47] Hauert C (2010). Replicator dynamics of reward & reputation in public goods games. J. Theor. Biol..

[48] Ohtsuki H, Iwasa Y (2004). How should we define goodness?–reputation dynamics in indirect reciprocity. J. Theor. Biol..

[49] Semmann D, Krambeck H-J, Milinski M (2004). Strategic investment in reputation. Behav. Ecol. Sociobiol..

[50] Li Y (2014). The evolution of reputation-based partner-switching behaviors with a cost. Sci. Rep..

[51] Fehr E (2004). Human behaviour: don’t lose your reputation. Nature.

[52] Huang K, Zheng X, Li Z, Yang Y (2015). Understanding cooperative behavior based on the coevolution of game strategy and link weight. Sci. Rep..

[53] Chen W, Wu T, Li Z, Wang L (2015). Coevolution of aspirations and cooperation in spatial prisoner’s dilemma game. J. Stat. Mech..

